# Emotional Dysregulation and Trauma Symptoms Mediate the Relationship Between Childhood Abuse and Nonsuicidal Self-Injury in Adolescents

**DOI:** 10.3389/fpsyt.2022.897081

**Published:** 2022-07-28

**Authors:** Hedvig Andersson, Erik Aspeqvist, Örjan Dahlström, Carl Göran Svedin, Linda S. Jonsson, Åsa Landberg, Maria Zetterqvist

**Affiliations:** ^1^Center for Social and Affective Neuroscience, Department of Biomedical and Clinical Sciences, Linköping University, Linköping, Sweden; ^2^Department of Behavioural Sciences and Learning, Linköping University, Linköping, Sweden; ^3^Department of Social Sciences, Marie Cederschiöld University, Stockholm, Sweden; ^4^Department of Child and Adolescent Psychiatry, Linköping University Hospital, Linköping, Sweden

**Keywords:** emotional dysregulation, childhood abuse, sexual abuse, physical abuse, emotional abuse, nonsuicidal self-injury, trauma, mediation

## Abstract

**Background:**

Nonsuicidal self-injury (NSSI) is common in adolescents. Emotion dysregulation has been identified as a core mechanism in the development and maintenance of NSSI and it is therefore an important target when addressing NSSI. The pathogenic connection between different kinds of childhood abuse, difficulties in emotion regulation and NSSI needs further investigation. The objective of this study was to examine whether difficulties with emotion regulation and trauma symptoms, separately and together, mediate the relationships between sexual, physical and emotional abuse and NSSI.

**Method:**

Cross-sectional data was collected from 3,169 adolescent high-school students aged 16–19 years (*M* = 18.12, *SD* = 0.45). Data from self-reported experiences of childhood abuse, current difficulties with emotion regulation (measured with the Difficulties with Emotion Regulation Scale, DERS-16) and trauma symptoms (measured with the Trauma Symptom Checklist for Children, TSCC), and NSSI were collected. Structural Equation Modeling (SEM) was used to test the proposed relationships between variables.

**Results:**

The prevalence of life-time NSSI was 27.4%. Prevalence of reported childhood abuse was 9.2, 17.5, and 18.0% for sexual, physical, and emotional abuse, respectively. Childhood abuse, difficulties with emotion regulation and trauma symptoms exhibited significant positive associations with NSSI in adolescents. Emotional dysregulation and trauma symptoms were both found to mediate the relationship between childhood abuse and NSSI. Latent variable models were found to fit data well.

**Conclusion:**

Results indicate that increased levels of emotional dysregulation and trauma symptoms in relation to childhood abuse contribute to the increased risk of NSSI. Further, results point to some aspects of emotional dysregulation and trauma symptoms being more important in this regard. Clinical implications are discussed.

## Introduction

Non-suicidal self-injury (NSSI) is defined as the direct and deliberate destruction of one’s own body tissue without suicidal intent (1). Different methods include hitting, cutting and burning oneself (2). There is a curvilinear relationship between age and NSSI (3), and NSSI is most common in adolescents, with a community prevalence around 17% (4–6). Recent data even suggest a potential increase in adolescent NSSI during the COVID-19 pandemic (7), making it a highly relevant and concerning behavior. NSSI is associated with negative long-term consequences such as mental health issues, lower self-esteem, social isolation and increased risk for suicide attempts (8). Emotion regulation (ER), or rather dysregulation, has been identified as a core mechanism in the development and maintenance of NSSI. A meta-analysis revealed that greater difficulties with ER were associated with greater likelihood to engage in NSSI (9). Especially limited access to ER strategies, non-acceptance of emotional responses, difficulties with impulse control and difficulties engaging in goal-directed behavior when experiencing negative emotions, according to the definition of emotion dysregulation (ED) by Gratz and Roemer (10), increased the risk of NSSI. There is ample empirical support that NSSI regulates emotion (11) and engaging in NSSI with the aim of changing an unwanted internal state has consistently been reported as the most common reason why an individual engages in NSSI (12). That NSSI is performed to change unwanted emotional experiences has also been emphasized in the proposed criteria of the suggested NSSI disorder diagnosis (13).

How NSSI and difficulties with ER develop during childhood and adolescence, specifically in relation to early detrimental childhood experiences, such as different kinds of abuse, is an area in need of further investigation.

### Nonsuicidal Self-Injury and Childhood Maltreatment

In order to detect, prevent and treat NSSI, research has focused on identifying potential risk factors for the behavior. Childhood maltreatment is one risk factor that has received considerable attention in relation to NSSI. For instance, childhood maltreatment (both maternal and paternal) is associated with the presence of NSSI (14), and an increased risk of later NSSI (15, 16). People with a lifetime history of NSSI report significantly more experiences of childhood maltreatment than people without a lifetime history of NSSI (17).

Even though there is an association between childhood maltreatment and NSSI, the relationship is complex. One factor contributing to the complexity is that the association seems to be affected by which type of maltreatment is being measured (emotional neglect and abuse, physical neglect, and abuse or sexual abuse). Some earlier studies (17) have reported that emotional abuse and neglect were the only types of childhood maltreatment that were directly associated with NSSI, whereas physical and sexual abuse were not. Others (18) have reported associations between physical neglect and NSSI, and between sexual abuse and NSSI. Weierich and Nock (19), in contrast, found a relationship only between sexual abuse and NSSI, and not between non-sexual abuse and NSSI. Experience of sexual abuse in relation to NSSI was also stressed in the review by Serafini et al. (15). Taken together, the research regarding the relationship between different subtypes of childhood maltreatment and NSSI is somewhat inconclusive and further research is needed.

Another factor that adds to the complexity, is that the relationship between childhood maltreatment, such as sexual abuse, and NSSI becomes much less pronounced when variables such as family environment, depression, dissociation and alexithymia are controlled for (20). The relationship between childhood maltreatment and NSSI also depends on the sample examined. Liu et al. (21), for instance, found that the relationship between childhood maltreatment and NSSI was stronger in non-clinical samples. Moderating factors such as gender (15) and how childhood maltreatment and NSSI is measured also play a role.

### Childhood Maltreatment, Emotion Dysregulation, Trauma Symptoms, and NSSI

How childhood maltreatment is related to NSSI, and whether the relationship is mediated through a third variable, has been investigated in earlier research. Potential mediators that have been examined are for instance symptoms of post-traumatic stress disorder (PTSD) (19), self-criticism (18), anxiety and depressive symptoms (17), self-blame, alexithymia, and dissociation (16), and also difficulties with ER (14, 22). Different types of difficulties with ER mediate the relationship between different types of childhood maltreatment and NSSI. Swannell et al. (16) found alexithymia, the inability to identify and describe emotions, to mediate the relationship between childhood maltreatment and NSSI in women, and this was especially salient for physical abuse. Guérin-Marion et al. (14) found that ER difficulties, measured as limited access to ER strategies and emotional clarity, to mediate the relationship between childhood maltreatment (measured as neglect, physical and psychological abuse) and NSSI. Emotional expressivity is another aspect of ER that has been identified to mediate the relationship between specifically emotional abuse and NSSI (22). Another study (23) found ED to mediate the relationship between both physical and emotional childhood maltreatment and NSSI frequency. Sexual abuse, however, was not associated with ED in this study (23). Further considering difficulties with ER, Brown et al. (17) found emotional maltreatment, in terms of abuse and neglect, to be directly associated with NSSI. In their study no other type of childhood maltreatment (sexual abuse, physical abuse or neglect) was directly associated with NSSI. These types of abuse were fully mediated through symptoms of depression and anxiety (17). Other studies (24, 25) confirm the relationship between emotional maltreatment and NSSI, which together emphasize the central role of emotional abuse and ED in relation to NSSI.

Existing research thus shows mixed results regarding the relationship between different types of childhood maltreatment and NSSI and whether the relationship is direct or rather indirect, and explained by a third variable. Emotional abuse seems to be of particular interest considering the potential risk of developing difficulties with ER, and NSSI.

Another potential mediating factor is PTSD or trauma symptoms (TS). Weierich and Nock (19), for instance, found reexperiencing and numbing/avoidance symptoms of PTSD to mediate the relationship between sexual abuse and NSSI (19). PTSD or TS evoked after experiencing childhood maltreatment may also be connected to experiencing difficulties with ER. Numbing, for example, may cause discomfort of feeling “empty” and therefore trigger dysfunctional behaviors to generate feelings, to avoiding feeling numb, which is a commonly reported function of NSSI (12). Both TS and difficulties with ER are therefore of particular interest when investigating the relationship between childhood maltreatment and NSSI.

Taken together, the pathogenic connection between different kinds of childhood abuse, difficulties with ER, TS and NSSI needs further investigation. The objective of this study was to examine whether difficulties with ER and TS, separately and together, mediate the relationship between childhood abuse (sexual, physical and emotional abuse) and NSSI.

It was hypothesized that there would be a significant positive relationship between childhood abuse and NSSI, especially between emotional abuse and NSSI, and that difficulties with ER and TS would mediate this relationship.

## Materials and Methods

### Procedure

Data was collected online in classrooms and during home studies in a representative sample of third year students in Swedish high schools during 2020–2021.

The schools were selected based on information from the national school register and stratified to represent a normal population of third year Swedish high school students regarding school size and study program, and were then randomized. Principals of selected schools were informed by e-mail and then contacted by phone. If no contact was established after three attempts the school was excluded. Participation was voluntary, if the principal agreed a date was set for gathering information through an online questionnaire that was filled in during lecture time in the presence of field workers. Students received written information, were informed that study participation was voluntary and gave informed consent to participate by answering the questionnaire. According to the Swedish Ethical Review Act (26) (SFS 2003:460), active consent is not required from parents when adolescents are 15 years of age or older. The survey was anonymous. To ensure confidentiality when participants filled in the questionnaire in school, the participants used their own computer and were placed with sufficient distance to others. Specially appointed staff was present. If participants filled in the questionnaire outside of school a unique identity was created, which was not revealed to anyone. Mandatory reporting was not possible due to the anonymity of the survey. To assure the students safety and wellbeing, participants received written information about where to turn for help and support, if needed.

A total of 210 schools with 7,752 students were selected and of these 110 schools with 3,286 students competed the questionnaire. Four non-serious questionnaires were excluded rendering 3,282 students and yielding a response rate of 42.3%. Due to the COVID-19-pandemic and the periodic closure of Swedish high schools the data collection was interrupted, extended, and changed to an online version that was possible to get access to from other places than the classroom. This resulted in three periods of data collection during 2020 and 2021, the spring of 2020 (*n* = 1,195), the autumn of 2020 (*n* = 737), and the spring of 2021 (*n* = 1,350). The response rate differed between the three periods of data collection. During the data collection in classrooms the response rate was 57.1 and 58.4%, while the response rate during the third period was 30.7%. Of the 3,282 participants, 114 were older than 19 years and were therefore excluded in the present study, following the United Nations definition of adolescence as the period between 13 and 19 years (27), which resulted in 3,169 adolescent participants in the present study.

The study was approved by the Swedish Ethical Review Authority (2019-05013-31, 2020-03611, 2020-06556).

### Participants

In the present study, 3,169 adolescent high-school students aged 16–19 years (*M* = 18.1, *SD* = 0.5) were included. Of these, 1,383 (43.6%) were male, 1,761 (55.6%) were female and 25 (0.8%) identified as non-binary. For participants’ background information and demographic data, see Table 1.

**TABLE 1:** Background and sociodemographic data for the adolescent high-school sample (*n* = 3,169).

	*n* (%)
**Gender**	
Boy	1,383 (43.6)
Girl	1,761 (55.6)
Non-binary identification	25 (0.8)
**Age** (*M*, *SD*)	18.1 (0.5)
**Study program**	
Theoretical	2,313 (73.0)
Vocational	757 (23.9)
Individual (lacks formal merits for high-school)	99 (3.1)
**Parents’ occupation**	
Fathers working	2,768 (87.3)
Mothers working	2,798 (88.3)
**Parents’ education**	
Fathers with university/college education	1,250 (39.4)
Mothers with university/college education	1,738 (54.8)
**Perception of financial situation in the family**	
Good	2,396 (75.6)
Neither good nor bad	612 (19.3)
Poor	111 (3.5)
Do not know	50 (1.6)
**Country of origin**	
Adolescents born in Sweden	2,880 (90.9)
Fathers born in Sweden	2,573 (81.2)
Mothers born in Sweden	2,545 (80.3)
**Living situation**	
With both parents	2,009 (63.4)
Alternating between both parents	362 (11.4)
With one parent with or without new partner	630 (19.9)
Alone or with siblings or partner	150 (4.7)
In foster care or institution	18 (0.6)

### Measures

The questionnaire used was a slightly modified version of a questionnaire used in three previous studies carried out in 2004, 2009 and 2014 (28–30). This study emanates from the survey “Young people, sex and the internet after #metoo” (31) and the questionnaire comprised 110 main questions concerning socio-demographic background, experiences of abuse, and risk behaviors. In addition, four standardized self-report instruments measuring psychosocial health, difficulties with emotional regulation, resilience and feelings of guilt and shame were used.

In the present study questions relating to sociodemographic background, NSSI, experiences of abuse, TS and difficulties with ER were used.

#### Demographic Information

Demographic questions were created for the purpose of the study assessing characteristics such as gender, type of study program (theoretical education programs, e.g., science, social studies, preparing for college/university; vocational programs, e.g., motor mechanics, electronics, hairdressing, preparing for a trade; individual programs for students who lack formal merits for high-school), parents’ occupation and education, perception of family’s economy, own and parents’ immigrant background and living situation. Adolescents self-reported demographic information in fixed answer categories (Table 1).

#### Nonsuicidal Self-Injury

Life-time prevalence of NSSI was assessed with the NSSI-item from the Self-Injurious Thoughts and Behaviors Interview [SITBI; (32)], short-form and self-report version: *“Have you ever actually engaged in non-suicidal self-injury (NSSI; that is, purposely hurt yourself without wanting to die, for example by cutting or burning)?”*

#### Childhood Maltreatement

Emotional and physical abuse were measured with an introductory single-item question: “*Do you have experience before the age of 18 that an adult has done any of the following to you?”*. Several examples were given of different kinds of emotional and physical abuse and participants could respond “never,” “on single occasions,” or “on several occasions.”

##### Emotional Abuse

A dichotomous variable of emotional abuse was constructed where emotional abuse was defined as having experience of being insulted (called worthless, stupid, ugly) or being treated like you didn’t exist on several occasions; or locked in to cellar, closet or similar; being locked out of your home; threatened to hit or harm you on single or several occasions.

##### Physical Abuse

A dichotomous variable of physical abuse was constructed where physical abuse was defined as having experience of being pulled by the hair or ear; slapped with hand; punched with hand or fist; kicked; burnt or scalded; pressed on neck/throat; hit with cain, belt or ruler or other; threatened or hurt you with knife or gun; hurt you with knife on single or several occasions.

##### Sexual Abuse

An introductory statement: “People can be pressured, convinced or forced to sexual acts that they cannot defend yourself against. Have you ever been exposed to any of the following?” was followed by different examples of sexual abuse with response alternatives “yes” or “no.” A dichotomous variable of sexual abuse was constructed where sexual abuse was defined as having experience of either penetrating sexual, oral or anal abuse. Only adolescents who reported that the sexual abuse had occurred before the age of 18 were included in the analysis.

##### Childhood Abuse

A dichotomous total childhood abuse variable was also created based on self-reported experiences of either emotional, physical or sexual abuse or no-abuse experience.

#### Trauma Symptoms

Trauma symptoms were measured using the Trauma Symptom Checklist for Children [TSCC; (33)]. The questionnaire includes 54 questions that can be divided into six categories; anxiety, depression, post-traumatic stress, sexual concerns, dissociation and anger. Response options are “never,” “sometimes,” “often,” and “almost all of the time” and scores range from 0 to 162 for the total scale. Higher scores indicate higher levels of trauma symptoms. When the instrument was psychometrically evaluated (33), Cronbach’s alpha was 0.84. The Swedish translation by Nilsson et al. (34) was used on the current sample. Cronbach’s alpha in the present sample was 0.96 for the full instrument, and 0.90 for subscale depression, 0.84 for anxiety, 0.88 for post-traumatic stress, 0.87 for dissociation, 0.85 for anger, and 0.82 for sexual concerns, indicating good to excellent internal consistency for all subscales and total scale.

#### Difficulties With Emotion Regulation

Difficulties with ER were measured using the Difficulties with Emotion Regulation Scale, 16-item version [DERS-16; (35)]. DERS-16 is a brief version of the 36-item original DERS version (10). DERS-16 consists of 16 items rated on a five-point Likert scale from “almost never” to “almost always.” Scores range from 16 to 80, where higher scores indicate more difficulties with regulating emotions. Cronbach’s alpha in the present sample was 0.95 for the total scale, indicating excellent internal consistency. DERS-16 includes five subscales: Nonacceptance (0.82), Goals (0.89), Impulse (0.90), Strategies (0.89), and Clarity (0.83), which all separately indicated good to excellent internal consistency in the present sample.

### Data Analysis

Data were analyzed with descriptive statistics using frequencies, percentages, mean values and standard deviations, and cross-tabulation with chi-square and odds ratio for categorical data, and independent samples t-test for group comparisons of continuous data. Internal consistency was assessed using Cronbach’s alpha (α) for the self-report measures. Statistical analyses were performed using the SPSS 28.0 software package (SPSS Inc, Chicago, IL), the R software package (36) and RStudio IDE (37). For Structural Equation Modeling (SEM), the lavaan package (38) was employed.

Mediation effects were investigated using a series of structural equation models. The different subtypes of abuse were specified as independent variables and NSSI as outcome. Mediation was evaluated using DERS-16 and TSCC sum scores as well as sub-scale scores.

To examine whether the hypothesized mediation effects could be represented as relationships between latent constructs reflected in sample data latent variable models were constructed. As several indicators were dichotomous, and as non-normal distribution could be assumed, models were fitted using the WLSMV estimator in lavaan (39, 40). Models were evaluated by means of fit indices Comparative Fit Index (CFI), Tucker Lewis Index (TLI), Standardized Root Mean Square Residual (SRMR), and Root Mean Square Error of Approximation (RMSEA). Chi-square results could a priori be assumed not to be useful given the large sample size (41). Robust (scaled) fit indices were used and, following Hu and Bentler (42), cut-off values for “relatively good fit” were set at “close to or under” 0.06 for RMSEA, “close to or over” 0.95 for TLI and CFI and “close to or under” 0.08 for SRMR. Correlation and covariance matrices available in the Appendix.

## Results

The lifetime prevalence of NSSI amounted to 27.4%. Childhood emotional abuse from an adult was reported by 18.0% of the participants, childhood physical abuse from an adult was reported by 17.5% and childhood sexual abuse by 9.2%. A history of childhood emotional (34.8%), physical (29.5%), and sexual abuse (20.9%) was significantly more commonly reported in those with NSSI compared to those without NSSI (11.8, 12.9, and 4.6%, respectively, all *p* < 0.001), although the effects sizes were small to medium (φ = 0.268, φ = 0.196, and φ = 0.253). Of those with NSSI, 51.2% had experienced at least one form of abuse, compared to 21.6% of those without NSSI, *p* < 0.001. Also, total scores on both DERS-16 and TSCC were significantly higher (*p* < 0.001) in those with NSSI (*M* = 48.1, *SD* = 15.5; *M* = 51.0, *SD* = 24.9) compared to those without (*M* = 32.2, *SD* = 13.4; *M* = 26.6, *SD* = 18.8). See Table 2.

**TABLE 2:** Frequencies and percentages of lifetime prevalence of childhood emotional, physical, sexual abuse in adolescents with and without NSSI, and means and standard deviations for trauma symptoms and difficulties with emotion regulation.

	Total sample *n* = 3,168* *n* (%)	No NSSI *n* = 2,281* n (%)	NSSI *n* = 867* *n* (%)	Statistics
Emotional abuse	571 (18.0)	267 (11.8)	302 (34.8)	χ ^2^ (1, *n* = 3148*) = 226.92, *p* < 0.001, φ = 0.268
Physical abuse	553 (17.5)	294 (12.9)	256 (29.5)	χ ^2^ (1, *n* = 3148*) = 120.60, *p* < 0.001, φ = 0.196
Sexual abuse	292 (9.2)	105 (4.6)	181 (20.9)	χ ^2^ (1, *n* = 3148*) = 201.42, *p* < 0.001, φ = 0.253
Any abuse	947 (29.9)	493 (21.6)	444 (51.2)	χ ^2^ (1, *n* = 3148*) = 263.25, *p* < 0.001, φ = 0.289
DERS-16 (*m*, *sd*)	36.5 (15.7)	32.2 (13.4)	48.1 (15.5)	*t* (3146) = 28.48, *p* < 0.001
TSCC (*m*, *sd*)	33.3 (23.4)	26.6 (18.8)	51.0 (24.9)	*t* (3146) = 29.65, *p* < 0.001

NSSI, nonsuicidal self-injury; DERS-16, Difficulties With Emotion Regulation Scale, 16-item version; TSCC, Trauma Symptoms Checklist for Children. *Missing data on NSSI status for 21 adolescents.

In the subgroups reporting abuse, NSSI was also more prevalent. Among adolescents reporting emotional abuse, 58.9% reported that they had self-injured without suicidal intent. The prevalence of NSSI in the groups reporting physical and sexual abuse were, 46.3 and 62.0%, respectively. Among those reporting history of at least one form of abuse, the prevalence was 46.9%. In the group not reporting abuse, the prevalence of NSSI was 19.2%. The risk of ever having engaged in NSSI was significantly higher with a history of abuse (for emotional abuse OR 4.03, 95% CI 3.34–4.87; for physical abuse OR 2.83, 95% CI 2.34–3.43; for sexual abuse OR 5.47, 95% CI 4.24–7.06; for any abuse OR 3.81, 95% CI 3.22–4.50).

**FIGURE 1:**
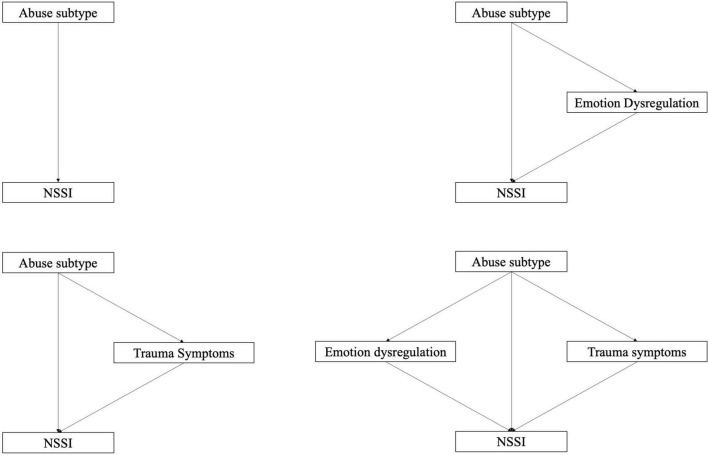
Models 1a–d: Modeling direct effect, single and parallel mediation.

Mediation was investigated using a series of models with ED and TS as mediators, by themselves and in parallel, using all three subtypes of abuse as independent variables (Model 1A-D; see Figure 1). Both ED and TS, by themselves, turned out to partially mediate the relationship between both abuse (emotional, physical as well as sexual) and NSSI (see Table 3).

**TABLE 3:** Effects with and without mediators (models 1a–c).

Independent	Mediator	Direct effect	Mediated effect
Emotional abuse	–	0.31***	–
Emotional abuse	ED	0.17***	0.14***
Emotional abuse	TS	0.11***	0.20***
Physical abuse	–	0.23***	
Physical abuse	ED	0.12***	0.12***
Physical abuse	TS	0.07***	0.17***
Sexual abuse	–	0.29***	
Sexual abuse	ED	0.18***	0.11***
Sexual abuse	TS	0.13***	0.16***

*Reported numbers are estimated standardized coefficients representing the total effect of the mediating path.*

***p < 0.001; ns, not significant.

Combined in parallel, in Model 1d, ED and TS mediated the relationship completely, regardless of abuse subtype (the direct effect became non-significant except in the case of physical abuse where it was significant but small and negative; see Table 4).

**TABLE 4:** Effects with and without parallel mediation (model 1d).

	Direct effect	Parallel mediation model			
Abuse subtype		Direct effect	Emotion dysregulation	Trauma symptoms	Mediation total
Emotional abuse	0.31***	–0.03 (ns)	0.14***	0.20***	0.34***
Physical abuse	0.23***	–0.05*	0.12***	0.17***	0.29***
Childhood sexual abuse	0.29***	0.02 (ns)	0.11***	0.16***	0.27***

*Reported numbers are estimated standardized coefficients representing the total effect of the mediating path.*

***p < 0.001; *p < 0.05; ns, not significant.

**FIGURE 2:**
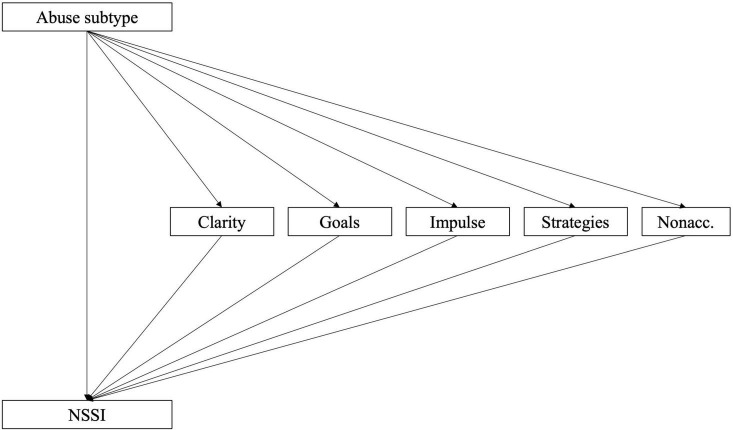
Model 2a: Sub-factors of DERS-16 as mediators of the relationship between childhood abuse and NSSI.

**FIGURE 3:**
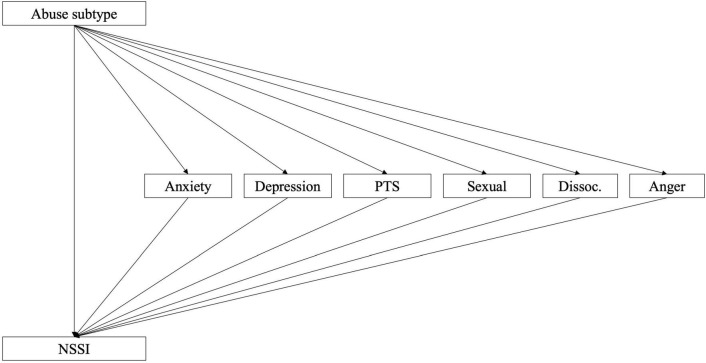
Model 2b: Sub-factors of TSCC as mediators of the relationship between childhood abuse and NSSI.

Follow-up analysis to investigate the relative strengths of different aspects of ED and trauma symptom factors (i.e., sub-scales of DERS-16 and TSCC) as mediators revealed that all factors partially mediated the effect using the three abuse subtypes (emotional, physical, and sexual) as independent variables. In a further step, when ED factors were modeled as parallel mediators and allowed to correlate (model 2a; see Figure 2), results echoed those of model 1b (see Table 5). The factor Strategies was found to be the most important mediator (see Table 5). Nonacceptance, Clarity, and Goals carried smaller but significant mediation effects, while the Impulse factor was found not to have a mediating effect. The pattern was similar regardless of abuse subtype used as independent variable. Similar results were found when TS factors were modeled as parallel mediators (model 2b; Figure 3 and Table 6). In this case, the Depression and PTSD Symptoms factors were the most important mediators (see Table 6). Anxiety carried a significant but small and negative mediation effect, while the effect of Sexual Concerns was found to be non-significant. The factor Dissociation was significant only when the effect mediated was that of emotional or physical abuse, while the factor Anger was significant only for sexual abuse.

**TABLE 5:** Effects with and without mediation (Models 1a and 2a).

	Direct effect (Model 1a)	Mediation (Model 2a)						
Abuse subtype		Direct effect	Clarity	Goals	Impulse	Strategies	Nonacceptance	Mediation total
Emotional	0.31***	0.17*** [0.129, 0.207]	0.02** [0.008, 0.029]	0.02* [0.001, 0.032]	–0.01 ns [–0.02, 0.007]	0.08*** [0.06, 0.109]	0.03*** [0.014, 0.048]	0.14*** [0.126, 0.162]
Physical	0.23***	0.12*** [0.079, 0.157]	0.02** [0.006, 0.025]	0.01* [0.001, 0.026]	–0.01 ns [–0.017, 0.007]	0.07*** [0.047, 0.087]	0.03*** [0.012, 0.040]	0.12*** [0.098, 0.135]
Sexual	0.29***	0.18*** [0.141, 0.218]	0.01** [0.006, 0.023]	0.01* [0.000, 0.024]	–0.01 ns [–0.017, 0.006]	0.07*** [0.045, 0.084]	0.02*** [0.011, 0.036]	0.11*** [0.091, 0.126]

*Reported numbers are estimated standardized coefficients representing the total effect of the mediating path, together with 95% confidence intervals.*

***p < 0.001; **p < 0.01; *p < 0.05; ns, not significant.

**TABLE 6:** Effects with and without mediation (model 2b).

	Direct effect (model 1a)	Mediation (Model 2b)							
Abuse subtype		Direct effect	Anx	Dep	PTS	SC	Dis	Ang	Combined
Emotional	0.31***	0.10*** [0.059, 0.139]	–0.02** [–0.042, –0.006]	0.13*** [0.111, 0.157]	0.05*** [0.023, 0.074]	–0.01 ns [–0.016, 0.004]	0.05*** [0.027, 0.068]	0.01 ns [–0.005, 0.031]	0.21*** [0.192, 0.233]
Physical	0.23***	0.07** [0.030, 0.108]	–0.02** [–0.035, –0.007]	0.10*** [0.076, 0.114]	0.04*** [0.017, 0.056]	–0.01 ns [–0.017, 0.002]	0.05*** [0.036, 0.069]	0.01 ns [–0.004, 0.025]	0.17*** [0.146, 0.186]
Sexual	0.29***	0.13*** [0.090, 0.168]	–0.03 ** [–0.042, –0.008]	0.11*** [0.093, 0.132]	0.05*** [0.029, 0.075]	–0.01 ns [–0.014, 0.004]	0.01 ns [0.009, 0.024]	0.02** [0.007, 0.030]	0.16*** [0.141, 0.178]

*Reported numbers are estimated standardized coefficients representing the total effect of the mediating path together with 95% confidence intervals.*

***p < 0.001; **p < 0.01; ns, not significant.

**FIGURE 4:**
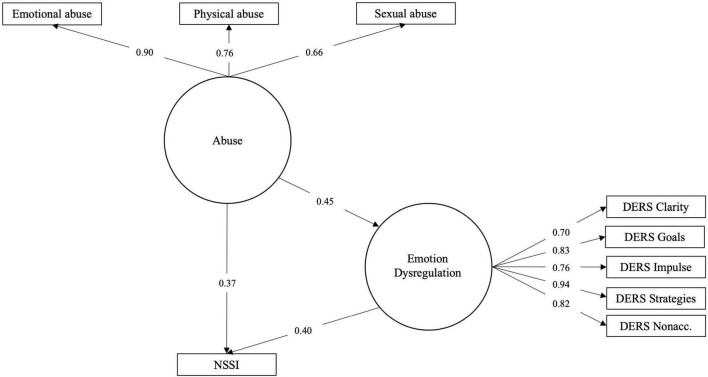
Model 3a: Latent variable model of abuse effect on NSSI risk mediated by ED.

**FIGURE 5:**
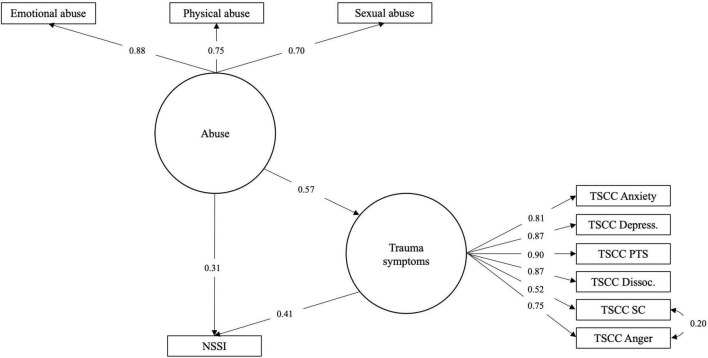
Model 3b: Latent variable model of abuse effect on NSSI risk mediated by TS.

**FIGURE 6:**
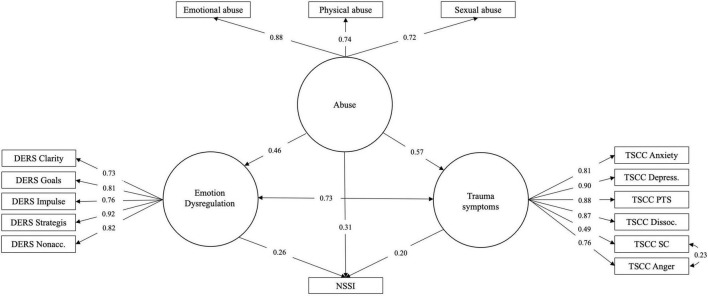
Model 3c: Latent variable model of abuse effect on NSSI risk mediated in parallel by ED and TS.

**FIGURE 7:**
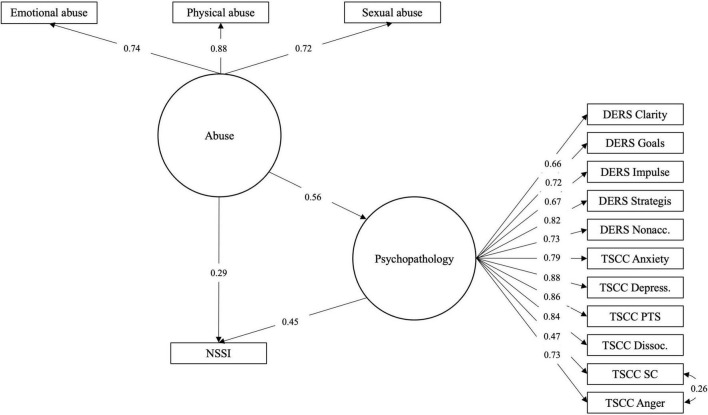
Model 3d: Latent variable model of abuse effect on NSSI risk mediated by general ED/TS factor.

With the objective to examine whether the effects previously indicated could be described as relationships between phenomena of a general character, SEM models were constructed using latent variables. Models were specified with abuse indicated by the three dichotomous survey items concerning sexual, physical and emotional abuse. ED and TS were specified as separate constructs indicated by the sub-scale/factor scores. The latent variables were then arranged in a standard mediation fashion (Models 3a for ED and 3b for TS; Figures 4, 5). Parallell mediation was modeled using both the ED and TS latent variables (Model 3c; Figure 6). Lastly, to examine to which degree ED and TS could be understood to represent a general psychopathology dimension, a model was fitted using both DERS-16 and TSCC sub-scale scores as indicating the same underlying construct, which was used as mediator between childhood abuse and NSSI (Model 3d; Figure 7). In order to obtain better fit, models including TSCC sub-factors (3b, c, and d) were adjusted, after inspection of modification indices, to estimate a correlation between the factors Anger and Sexual Concerns. Crossloadings of items belonging to these factors have been documented [e.g., (43)], and they were also found not to carry mediation (see Table 6).

Models 3a and 3c were found to fit the data well, while Model 3b was only approaching good fit (RMSEA slightly above cut-off; please see Table 7). This suggest that these are meaningful representations of how data are distributed. Mediation effects in these models were partial, similar to what had been found when evaluating mediation in models 1b and 1c. Model 3d was found not to fit data and is hence not a meaningful way to represent the relationships here investigated.

**TABLE 7:** Fit indices.

Model	df	RMSEA	CFI	TLI	SRMR
3a	25	0.046	0.967	0.952	0.043
3b	32	0.066	0.952	0.933	0.049
3c	84	0.058	0.928	0.911	0.042
3d	87	0.082	0.853	0.822	0.064

*See Figures 4–7.*

*Df, degrees of freedom; RMSEA, Root Mean Square Error of Approximation; CFI, Comparative Fit Index; TLI, Tucker-Lewis Index; SRMR, Standardized Root Mean Squared Residual.*

## Discussion

NSSI is a prevalent and serious mental-health concern in adolescents, and ED has been shown to be a core mechanism in the development and maintenance of NSSI. Childhood maltreatment is another factor that has received considerable attention in relation to NSSI. The nature of these associations are, however, not clear and this study contributes important information on this topic by examining the relationship between different types of childhood abuse and NSSI in a large community sample of Swedish adolescents, and to what degree difficulties with ER and TS mediate this relationship. NSSI was found to be highly prevalent in this sample, and experience of physical, emotional and sexual abuse were significantly more common in adolescents with NSSI, compared to those without NSSI, as were higher rates of TS and difficulties with ER. The results in the current study found a significant and positive association between all subtypes of childhood abuse and NSSI. As hypothesized, ED and TS was found to mediate this effect. With ED and TS as parallel mediators, the mediation was complete (the direct effect became non-significant). Thus, it is likely that increased levels of ED and TS in relation to childhood abuse explain part of the increased risk of NSSI.

The life-time prevalence of NSSI in this sample (27.4%) was higher than reported in prior studies of adolescents (4, 44). This potential increase in NSSI in community adolescents needs to be investigated further, to determine whether it is a stable or temporary trend due to some external factor, such as the COVID-19 pandemic (7).

Concerning rates of abuse in our sample, results were strikingly similar to an earlier study that examined prevalence rates of different kinds of abuse in a large Swedish high-school sample (45) and found self-reported rates of emotional abuse (16.6 vs. 18.0%), sexual abuse (10.2 vs, 9.2%) and a somewhat higher rate of physical abuse (26.5 vs. 17.5%) than the present study. However, in a later study, the prevalence of physical abuse was reported to have declined in Sweden from 18.2% in 2008 to 13.2% in 2017 (46), which is more consistent with our results. In the current study, more adolescents with a history of childhood abuse reported NSSI. Also, more adolescents with NSSI reported experiences of some form of childhood abuse, as well as more difficulties with emotion regulation (DERS-16) and trauma symptoms (TSCC) compared to those without NSSI. This difference between groups confirms results from earlier studies both in clinical (47) and community samples of adolescents (6), and implies that adolescents with NSSI are more burdened by adversitities and distress. Inversely, as indicated by odds ratios, the risk of having engaged in NSSI was significantly heightened for victims of childhood abuse.

The results further showed that all types of maltreatment investigated (sexual, physical and emotional abuse) were significantly associated with NSSI. As hypothesized, difficulties with ER and TS fully mediated the relationship between all subtypes of childhood maltreatment and NSSI. The relationship between abuse history and NSSI prevalence in the present sample can be described as an effect of abuse history on ED and TS, such that abuse seems to increase these symptoms. A history of physical abuse seems to be a little bit weaker than sexual and emotional abuse as a predictor.

Regarding the association between childhood abuse and NSSI, results in the current study are consistent with prior studies (14, 15, 17–19, 22), and also add to the research field by contributing data in a large community sample. Although some earlier studies only found an association between specific subtypes of maltreatment and NSSI (18, 19, 22), the current study found a significant relationship between all types of maltreatment and NSSI. One potential explanation for this difference may be that childhood maltreatment was measured in different ways. Some of the previous studies [for example (16, 17, 19, 22, 23)] used CTQ to measure childhood maltreatment, whereas the present study did not. The mediating effect of difficulties with ER is also consistent with previous studies (14, 16, 22, 23). Concerning the relationship between ED and NSSI, this confirms the important role of ED in relation to NSSI (9) and that one important function of NSSI is to regulate emotions and changing an unwanted internal state (12). Likewise, the mediating effect of TS is consistent with the earlier study by Weierich and Nock (19), even though TS were measured with a different questionnaire. TS was measured by TSCC in this study, which includes symptoms of depression, anxiety, posttraumatic stress, dissociation, sexual concerns and anger. The relationship between TS and NSSI could potentially be understood as a maladaptive way of trying to cope with or reduce these symptoms. Inspection of individual path coefficients indicate that the mediating effect of TS was somewhat stronger than that of ED. The important finding in the present study is the fact that ED and TS combined mediate the relationship between childhood maltreatment and NSSI, pointing to a pathway between childhood maltreatment and NSSI, via ED and TS.

In consistence with the results of Wolff et al. (9), the DERS-16 subscale Strategies seemed to be the most salient predictor of NSSI pointing to this aspect of ED as being important to address when working with NSSI in victims of childhood abuse. Strategies, in fact, carried more than half of the effect mediated by ED while Impulse was found not to be a significant mediator. Limited access to ER strategies has been found to mediate this association also in other studies (14), and the results of the current study contribute further information by confirming this association in a much larger sample. An interpretation of the mediation effect via Strategies could be that victims of childhood abuse to a higher degree lack adaptive ER strategies and thus are more likely to engage in NSSI instead as a way to regulate intense emotions. These results emphasize the role of focusing on specific ER skills in the treatment of NSSI.

Similarly, some factors, represented by TSCC subscales as separate mediators, turned out to have higher explanatory importance; the depression, post-traumatic stress and dissociation factors carried most of the mediated effect while anxiety, anger and sexual concerns were found to be weak and/or insignificant mediators, with some differences depending on abuse type. This is consistent with previous results (19), indicating that PTSD-symptoms mediated the relationship between childhood maltreatment and NSSI. Weierich and Nock (19) found reexperiencing and avoidance/numbing symptoms of PTSD to mediate the relationship, which may correspond to the subscales post-traumatic stress and dissociation in TSCC. Symptoms of depression have also previously been shown to be a risk factor for NSSI (48). The results from the current study emphasize the importance of screening for and specifically targeting symptoms of depression and post-traumatic stress as a means to reduce the need to engage in NSSI.

Structural equation modeling of these phenomena and their proposed relationships as latent variables proved to be meaningful, as the mediation effects could be demonstrated also in this way and as two of the models fit sample data adequately. In the earlier models, abuse sub-types were examined and compared as independent variables in separate mediation models, but in the SEM models, childhood abuse as a general construct indicated by three dichotomous items was found to be meaningful representation. Exploratory modeling of ED and TS as reflecting a single underlying construct (Model 3c) was unsatisfactory. This points to the fact that ED and TS, while related, are not overlapping constructs. Patterns found in mediation models without latent constructs were also repeated; emotional and sexual abuse seem to be more important predictors than physical abuse and the sub-factors used as indicators for the ED and TS latent variables are estimated with coefficients corresponding to their relative strengths as mediators.

The significant relationship found between all types of childhood abuse and difficulties with ER can be understood by applying the Biosocial Model (49). In the Biosocial Model, difficulties with ER are considered a result of an invalidating environment (adverse childhood experiences, for instance) together with individual biological vulnerability (49). Invalidating environments, such as parental criticism and lack of parental emotional support, for example, have previously been found to be associated with NSSI (50). The significant association found in the present study between difficulties with ER and NSSI, is in line with earlier conceptualizations of NSSI as a dysfunctional ER strategy (51). This is further strengthened by the fact that the subscale Strategies was found to be the most important mediating ED factor in the relationship between childhood abuse and NSSI. Childhood abuse may be associated with ED directly or through different psychiatric symptoms such as TS. TS, as mentioned above, may in turn be related to difficulties with ER, for example, avoiding feeling numb.

This study contributes important information on the relationship between childhood abuse, ED, TS and NSSI in adolescents. Strengths include using data from a large community sample, including several different types of abuse and validated measures of ER and TS. The study is not without limitations, however, and these need mentioning. First and foremost, all data are cross-sectional, which limits casual interpretations of pathways. ED and TS could cause an urge to engage in NSSI, but the relationship could also potentially be bidirectional with NSSI resulting in symptoms of depression and post-traumatic stress, for example, and limited access to emotion regulation skills. The reported abuse in the current study, however, occurred before the age of 18 and symptoms of emotional dysregulation and trauma were assessed as current experiences, whereas NSSI was assessed as life-time prevalence. Longitudinal studies are needed to further examine this pathogenic association. The reliability of the data regarding abuse history could also be limited as data is based on retrospective recall. Another limitation is that experience of abuse was not measured with a validated abuse measure, but instead questions created for the present study. Results from other large epidemiological studies of abuse rates in the same age group in Sweden confirm our prevalence rates, which, however, validated our prevalence rates. The response rate of 42.3% also needs mentioning. This was in part due to interruption of data collections due to COVID-19, and caution must thus be taken when results are generalized. In addition, the results cannot be generalized beyond community samples of older adolescents. Finally, as several of the variables in our sample were based on dichotomous survey items, there was no way to account for severity or frequency of abuse or NSSI severity. There is reason to believe that the effects and relationships here presented could be demonstrated in a better fashion given measures of a higher resolution.

The results emphasize the role of ED and TS in the relationship between childhood abuse and NSSI, which gives rise to several implications. It is important to screen for childhood abuse in adolescents with NSSI, and also to screen for NSSI in adolescents with a history of childhood abuse. The results also stress the need for ER skills training for children, adolescents and caregivers, especially when there is a history of childhood abuse. Both as a way of preventing future ED problems, and also to treat NSSI. Based on our results, it would be specifically meaningful to focus on decreasing symptoms of depression and post-traumatic stress, and also to increase strategies for ED.

In conclusion, the current study found a significant and positive relationship between all subtypes of childhood abuse and NSSI in a large community sample of Swedish adolescents. ED and TS were found to completely mediate this relationship. Results indicate that increased levels of emotional dysregulation and trauma symptoms in relation to childhood abuse contribute to the increased risk of NSSI.

## Data Availability Statement

The datasets presented in this article are not readily available because the dataset is not public. Requests to access the datasets should be directed to CS, carl-goran.svedin@mchs.se. Correlation and covariance matrices available in the Appendix.

## Ethics Statement

The studies involving human participants were reviewed and approved by the Swedish Ethical Review Authority (2019-05013-31, 2020-03611, and 2020-06556). Written informed consent from the participants’ legal guardian/next of kin was not required to participate in this study in accordance with the national legislation and the institutional requirements.

## Author Contributions

HA and EA analyzed the data. HA, EA, and MZ drafted the manuscript. All authors designed the research and read and provided feedback on the manuscript.

## Conflict of Interest

The authors declare that the research was conducted in the absence of any commercial or financial relationships that could be construed as a potential conflict of interest.

## Publisher’s Note

All claims expressed in this article are solely those of the authors and do not necessarily represent those of their affiliated organizations, or those of the publisher, the editors and the reviewers. Any product that may be evaluated in this article, or claim that may be made by its manufacturer, is not guaranteed or endorsed by the publisher.

## Appendix

### Correlation matrix

**Table:**

	Q33_DERS	Q33Clar	Q33Goal	Q33Imp	Q33Strat	Q33Nacc	Q55_TSCC	Q55Ang	Q55Dep	Q55PTS	Q55SC	Q55DIS	Q55Ilsk
Q33_DERS	1.00	0.74	0.87	0.82	0.95	0.86	0.72	0.62	0.74	0.65	0.33	0.65	0.59
Q33Clar	0.74	1.00	0.57	0.54	0.65	0.58	0.57	0.48	0.59	0.49	0.26	0.53	0.47
Q33Goal	0.87	0.57	1.00	0.67	0.78	0.65	0.60	0.55	0.61	0.56	0.28	0.54	0.46
Q33Imp	0.82	0.54	0.67	1.00	0.72	0.60	0.57	0.46	0.52	0.51	0.27	0.48	0.59
Q33Strat	0.95	0.65	0.78	0.72	1.00	0.79	0.70	0.59	0.74	0.63	0.31	0.62	0.54
Q33Nacc	0.86	0.58	0.65	0.60	0.79	1.00	0.63	0.54	0.66	0.56	0.30	0.57	0.46
Q55_TSCC	0.72	0.57	0.60	0.57	0.70	0.63	1.00	0.85	0.88	0.89	0.64	0.89	0.81
Q55Ang	0.62	0.48	0.55	0.46	0.59	0.54	0.85	1.00	0.75	0.80	0.40	0.71	0.60
Q55Dep	0.74	0.59	0.61	0.52	0.74	0.66	0.88	0.75	1.00	0.75	0.42	0.76	0.63
Q55PTS	0.65	0.49	0.56	0.51	0.63	0.56	0.89	0.80	0.75	1.00	0.46	0.78	0.64
Q55SC	0.33	0.26	0.28	0.27	0.31	0.30	0.64	0.40	0.42	0.46	1.00	0.49	0.50
Q55DIS	0.65	0.53	0.54	0.48	0.62	0.57	0.89	0.71	0.76	0.78	0.49	1.00	0.67
Q55Ilsk	0.59	0.47	0.46	0.59	0.54	0.46	0.81	0.60	0.63	0.64	0.50	0.67	1.00

### Covariance matrix

**Table:**

	child _sa	Q31_emo _ab	Q32_phys _ab	Q33 _DERS	Q33 Clar	Q33 Goal	Q33 Imp	Q33 Strat	Q33 Nacc	Q55 _TSCC	Q55 Ang	Q55 Dep	Q55 PTS	Q55 SC	Q55 DIS	Q55 Ilsk
child_sa	0.08	0.02	0.02	1.00	0.09	0.19	0.19	0.34	0.19	2.02	0.36	0.40	0.53	0.23	0.36	0.27
Q31_emo_ab	0.02	0.15	0.07	1.73	0.16	0.33	0.30	0.59	0.35	3.39	0.50	0.70	0.80	0.34	0.67	0.55
Q32_phys_ab	0.02	0.07	0.14	1.37	0.13	0.26	0.25	0.44	0.29	2.68	0.38	0.50	0.61	0.32	0.53	0.47
Q33_DERS	1.00	1.73	1.37	247.83	24.40	50.04	42.47	81.97	48.95	265.77	45.30	62.96	58.11	22.16	53.77	38.93
Q33Clar	0.09	0.16	0.13	24.40	4.42	4.38	3.74	7.46	4.39	28.04	4.69	6.71	5.91	2.29	5.90	4.13
Q33Goal	0.19	0.33	0.26	50.04	4.38	13.30	8.02	15.70	8.64	51.60	9.31	12.04	11.62	4.34	10.41	7.03
Q33Imp	0.19	0.30	0.25	42.47	3.74	8.02	10.71	12.93	7.07	43.45	6.97	9.20	9.46	3.72	8.37	8.09
Q33Strat	0.34	0.59	0.44	81.97	7.46	15.70	12.93	30.18	15.71	89.53	15.15	22.06	19.64	7.20	18.12	12.58
Q33Nacc	0.19	0.35	0.29	48.95	4.39	8.64	7.07	15.71	13.14	53.16	9.18	12.95	11.50	4.61	10.96	7.10
Q55_TSCC	2.02	3.39	2.68	265.77	28.04	51.60	43.45	89.53	53.16	548.11	93.55	111.75	119.02	64.30	110.39	79.65
Q55Ang	0.36	0.50	0.38	45.30	4.69	9.31	6.97	15.15	9.18	93.55	21.86	19.16	21.26	8.02	17.52	11.80
Q55Dep	0.40	0.70	0.50	62.96	6.71	12.04	9.20	22.06	12.95	111.75	19.16	29.61	23.20	9.64	21.87	14.43
Q55PTS	0.53	0.80	0.61	58.11	5.91	11.62	9.46	19.64	11.50	119.02	21.26	23.20	32.42	11.26	23.36	15.28
Q55SC	0.23	0.34	0.32	22.16	2.29	4.34	3.72	7.20	4.61	64.30	8.02	9.64	11.26	18.17	11.11	9.08
Q55DIS	0.36	0.67	0.53	53.77	5.90	10.41	8.37	18.12	10.96	110.39	17.52	21.87	23.36	11.11	28.01	15.08
Q55Ilsk	0.27	0.55	0.47	38.93	4.13	7.03	8.09	12.58	7.10	79.65	11.80	14.43	15.28	9.08	15.08	17.84
